# Molecular Mechanism of AtCAX3-Induced BPA Tolerance in Plants

**DOI:** 10.3390/plants15193042

**Published:** 2026-10-05

**Authors:** HyoCheol Shin, DongGwan Kim, JunHyun Lee, Kyung Hwa Kee, Hye Hyun Yoo, SeongBin Hwang

**Affiliations:** 1 Department of Molecular Biology, Sejong University, Seoul 05006, Republic of Korea; hyocheol2@wooree.co.kr (H.S.); kimdg@sejong.ac.kr (D.K.); 2 Department of Bioindustry and Bioresource Engineering, Sejong University, Seoul 05006, Republic of Korea; 3 Plant Engineering Research Institute, Sejong University, Seoul 05006, Republic of Korea; 4 College of Pharmacy, Hanyang University, Ansan 15588, Gyeonggi-do, Republic of Korea; wkwmdsk1106@naver.com (J.L.); khkee9202@gmail.com (K.H.K.); yoohh@hanyang.ac.kr (H.H.Y.)

**Keywords:** antioxidant enzyme, BPA, Ca, CAX3, ROS

## Abstract

Cation/H^+^ Exchangers (CAXs) are involved in the sequestration of cations such as Ca^2+^, Mn^2+^, Li^+^, Zn^2+^, and Cd^2+^ from the cytosol into the vacuole using proton gradients. Our previous studies demonstrated the involvement of AtCAX3 in Cd^2+^ tolerance. In this study, we investigated whether AtCAX3 is also involved in tolerance to the endocrine disruptor Bisphenol A (BPA). Furthermore, to examine the role of the autoinhibitory domain of AtCAX3 in BPA tolerance, the empty vector (pYES5), *AtCAX3*, Δ36-*AtCAX3* (deletion of the N-terminal regulatory region; NRR), and Δ90-*AtCAX3* (deletion of both the NRR and regulatory-dependent region; RDR) were overexpressed in *Saccharomyces cerevisiae* and the *atcax3* knockout mutant of *Arabidopsis thaliana*. In Arabidopsis, BPA tolerance, Ca^2+^ levels, and antioxidant enzyme activities were highest in Δ90-*AtCAX3*-overexpressing plants, followed by Δ36-*AtCAX3*, *AtCAX3*, Col-0, and *atcax3*. Conversely, ROS levels followed the opposite pattern. Interestingly, the expression levels of Ca^2+^ importer genes (*AtCNGC10* and *AtMCA1*) were increased, whereas those of Ca^2+^ exporter genes (*AtACA10*) were decreased in the order of Δ90-*AtCAX3* > Δ36-*AtCAX3* > *AtCAX3* > Col-0 > *atcax3*. These results suggest that elevated Ca^2+^ levels may contribute to ROS reduction. Taken together, deletion of the autoinhibitory domain of AtCAX3 enhances BPA tolerance by reducing BPA-induced ROS accumulation through increased Ca^2+^ accumulation and activation of antioxidant defense systems. Furthermore, enhanced Ca^2+^ homeostasis may interfere with the positive feedback loop between BPA-induced ROS generation and Ca^2+^ spikes. These findings can be applied to develop BPA phytoremediators and BPA-resilient crops.

## 1. Introduction

Plants are sessile organisms that cannot escape environmental stresses and are therefore exposed to various stress factors throughout their life cycle [1]. In particular, exposure to abiotic stresses, such as heavy metals and endocrine-disrupting chemicals, can disrupt cellular homeostasis, inhibit growth, and, in severe cases, lead to plant death [2].

The Food and Agriculture Organization (FAO) has categorized bisphenol A (BPA) as an emerging soil pollutant of concern [3]. BPA concentrations vary considerably among contaminated environments [4]. In soils, BPA concentrations have been reported to range from 2 to 14 μg/kg dry weight in the United States, while concentrations of up to 140 μg/kg and 48.7 μg/kg dry weight have been reported in Europe and Korea, respectively [5]. In agricultural soils, BPA concentrations range from 32 to 147 μg/kg in the United States [6] and from 1.6 to 30.2 μg/kg in Mexico [7]. Furthermore, BPA concentrations in leachates from hazardous waste landfills in Japan have been reported to range from 1.3 to 17,200 μg/L [8], whereas concentrations in streams and rivers are generally below 21 μg/L [9].

Bisphenol A (BPA; 2,2-bis(4-hydroxyphenyl)propane), a bisphenol derivative, is one of the most well-known endocrine-disrupting chemicals (EDCs) and can interfere with hormonal homeostasis. BPA is widely used in the production of plastics, electronic products, disposable containers, and medical devices, and has emerged as a major environmental pollutant. Despite growing environmental concerns, global demand for plastics continues to increase, with annual BPA production exceeding 2 million tons, thereby posing a potential threat to ecosystems and human health [10,11,12]. Owing to its hydrophobic nature, BPA tends to accumulate in plant roots following uptake and is poorly translocated to aerial tissues. Consequently, inhibition of root growth is one of the most common symptoms of BPA toxicity in plants [13,14]. BPA-induced phytotoxicity has been associated with excessive reactive oxygen species (ROS) generation, reduced biomass accumulation, and various physiological impairments in roots [15]. Furthermore, because BPA is structurally similar to estrogen [16], its accumulation in animals and humans may disrupt endocrine function and reproductive processes [17]. The effects of BPA on plants may vary markedly depending on the exposure concentration, with low concentrations potentially promoting growth and higher concentrations inducing phytotoxic effects.

High concentrations of BPA have been shown to induce ROS accumulation in various plant species, including soybean [18], cucumber [19], carrot [20], and *Syngonium podophyllum* [21]. In Arabidopsis, BPA-induced root growth inhibition has been attributed to excessive ROS accumulation and lipid peroxidation [22]. In addition, BPA-induced root growth inhibition and auxin accumulation are mediated by ROS in Arabidopsis [23].

Calcium ions (Ca^2+^) function as important secondary messengers in plant responses to environmental stresses [24]. Stress-induced ROS production is known to trigger transient increases in cytosolic Ca^2+^ levels, which subsequently activate downstream defense signaling pathways [25]. However, the molecular mechanisms regulating Ca^2+^ homeostasis during BPA stress remain largely unclear.

CAX (cation/H^+^ exchanger) proteins are vacuolar transporters that mediate the sequestration of cations such as Ca^2+^, Mn^2+^, Na^+^, Li^+^, and Cd^2+^ into the vacuole using proton gradients generated by H^+^ pumps [26,27,28]. Among the six CAX genes identified in *Arabidopsis thaliana*, AtCAX3 is a tonoplast-localized transporter involved in responses to ionic and pH stresses [29]. The activity of AtCAX3 is regulated by autoinhibitory domains, including the N-terminal regulatory region (NRR) and the regulatory-dependent region (RDR) [30].

Previous studies have demonstrated that CAX proteins contribute to plant tolerance against various abiotic stresses through regulation of Ca^2+^ homeostasis and ROS accumulation. Altered expression of CAX genes affects ROS levels and tolerance to stresses such as cadmium [29] and Ba^2+^ toxicity [31], salinity [32,33], waterlogging [34]. In addition, Ca^2+^ has been reported to enhance antioxidant enzyme activities under drought, heat, and arsenic stress conditions [35,36,37].

Previously, we demonstrated that AtCAX3 enhances Cd^2+^ tolerance through Ca^2+^-mediated reduction in ROS accumulation [38]. In the present study, we investigated the role of AtCAX3 and its autoinhibitory domains in BPA tolerance. To examine the effects of autoinhibition, two deletion constructs, Δ36-*AtCAX3* and Δ90-*AtCAX3*, lacking the NRR alone or both the NRR and RDR, respectively, were generated. Our results showed that overexpression of *AtCAX3*, Δ36*-AtCAX3*, and Δ90-*AtCAX3* significantly enhanced BPA tolerance by reducing ROS accumulation and increasing antioxidant defense activities and Ca^2+^ levels. In addition, BPA levels were enhanced in *AtCAX3*- and Δ36-*AtCAX3*-expressing plants. These findings suggest that AtCAX3 may contribute to phytoremediation of BPA-contaminated environments and provide new insights into the role of CAX proteins in plant abiotic stress responses.

## 2. Results

### 2.1. Overexpression of N-Terminal Truncated (Removal of Autoinhibitory Domain) AtCAX3 Enhances BPA Tolerance in Yeast

**Figure 1:**
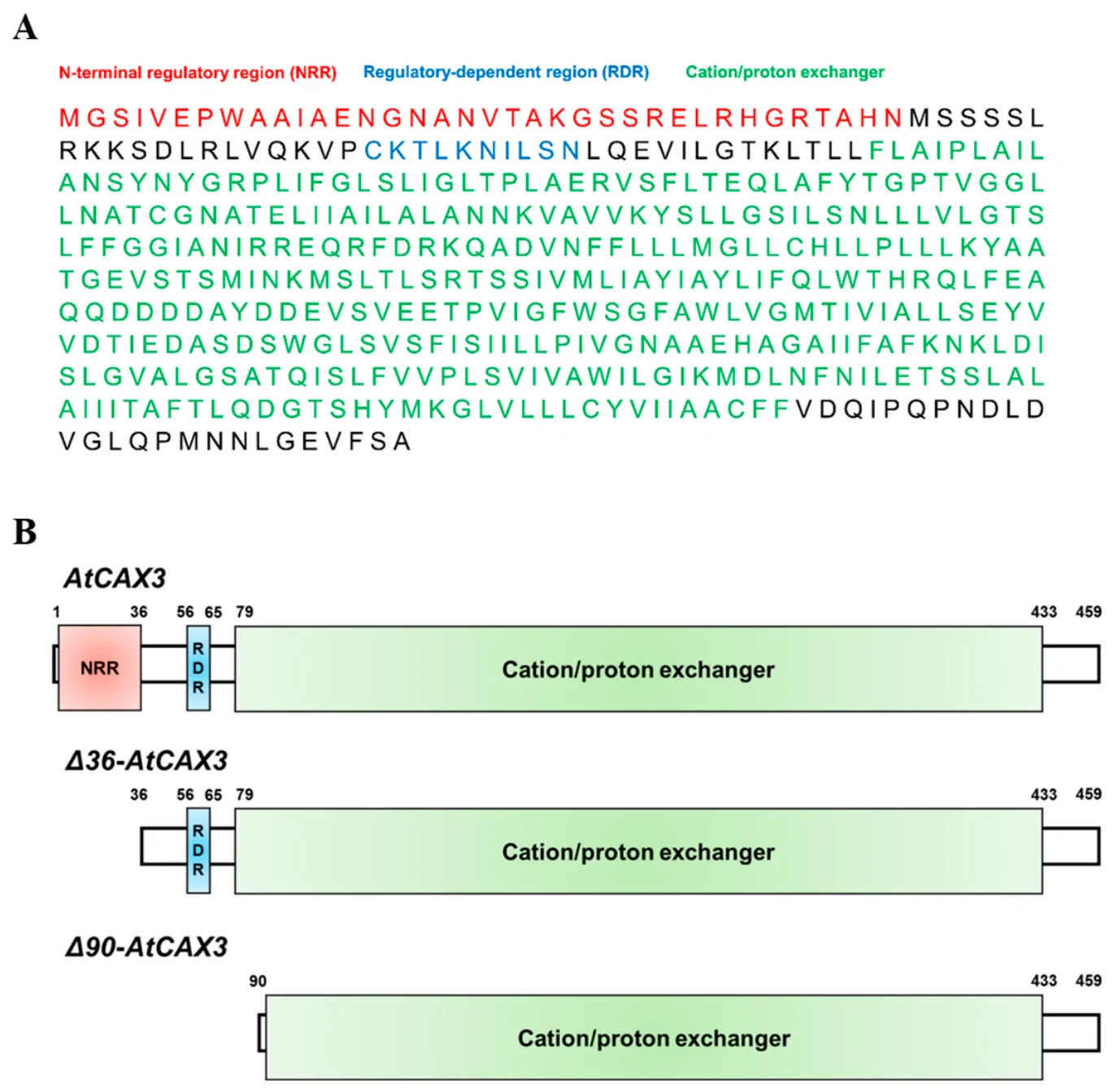
**Structure of AtCAX3 and N-terminal truncated constructs.** (**A**) Schematic representation of the AtCAX3 protein, including the N-terminal regulatory region (NRR), the regulatory-dependent region (RDR), and the cation/proton exchanger domain. (**B**) Diagram of various *AtCAX3* deletion constructs. In Δ36-AtCAX3, the first 36 amino acids comprising the NRR were deleted. In Δ90-AtCAX3, the first 90 amino acids, including both the NRR and RDR, were removed.

Previously, we expressed both full-length AtCAX3 and an N-terminal truncated form, Δ90-AtCAX3 (lacking the first 90 amino acids, including NRR and RDR domains), in K667 yeast. While expression of Δ90-AtCAX3 enhanced tolerance to Ca^2+^ and Cd, full-length AtCAX3 did not [38]. These results suggested that removal of the autoinhibitory domain activates AtCAX3, likely leading to increased vacuolar Ca^2+^ accumulation and enhanced tolerance to Ca^2+^ and Cd stress. To further investigate the role of AtCAX3 and its N-terminal truncated variants in BPA tolerance, two deletion constructs, Δ36-AtCAX3 (36-aa deletion lacking NRR) and Δ90-AtCAX3 (90-aa deletion lacking NRR and RDR), were generated (Figure 1).

**Figure 2:**
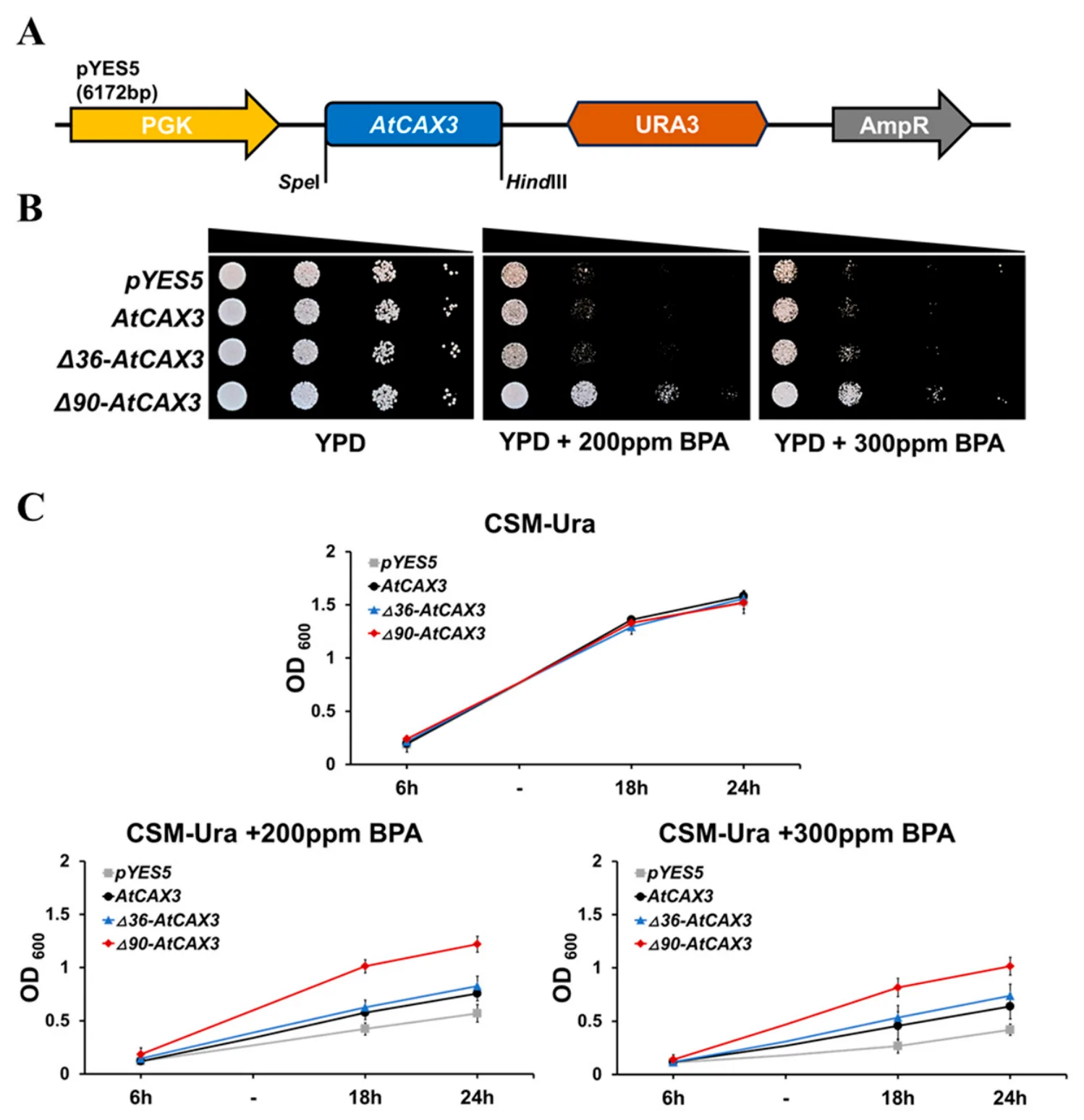
**BPA tolerance in yeast expressing full-length and N-terminally truncated AtCAX3.** (**A**) Map of the pYES5 vector containing the PGK promoter, URA3 selection marker, and ampicillin-resistance gene. AtCAX3 and truncated variants (Δ36 and Δ90) were subcloned into this vector. (**B**) Growth phenotypes of yeast (ATCC 4001531) expressing different AtCAX3 constructs. Cells were serially diluted and grown for 2 days on YPD medium supplemented with various concentrations of BPA. (**C**) Growth curves of yeast strains in liquid culture. All strains were cultured in 20 mL CSM-Ura liquid medium with indicated BPA concentrations at 30 °C. The initial optical density (OD_600_) was adjusted to 0.1, and growth was monitored at 6, 18, and 24 h. Data represent the mean of three independent experiments; error bars indicate standard error (SE).

These constructs, together with full-length AtCAX3, were transformed into yeast cells. On agar medium containing 200 and 300 ppm BPA (Figure 2B), yeast expressing Δ90-AtCAX3 exhibited the highest tolerance. Yeast expressing Δ36-AtCAX3 showed moderately enhanced tolerance compared with full-length AtCAX3 and the pYES5 vector control, which displayed similar levels of sensitivity. The observation that full-length AtCAX3 did not significantly enhance BPA tolerance is consistent with our previous findings for Cd and Ca^2+^ tolerance [38].

Growth patterns in liquid culture (Figure 2C) were generally consistent with those observed on agar medium. However, unlike the agar assay, expression of full-length AtCAX3 slightly increased BPA tolerance compared with the pYES5 control. This difference may be attributed to the different culture conditions used for YPD agar and CSM-Ura liquid cultures (see Section 4.4). Taken together, these results suggest that AtCAX3 contributes to BPA tolerance and that deletion of its autoinhibitory domains, particularly in Δ90 and Δ36, markedly enhances this effect, possibly through improved BPA stress adaptation.

### 2.2. Expression of AtCAX3 and Its Truncated Variants Reduces ROS Levels in Yeast

**Figure 3:**
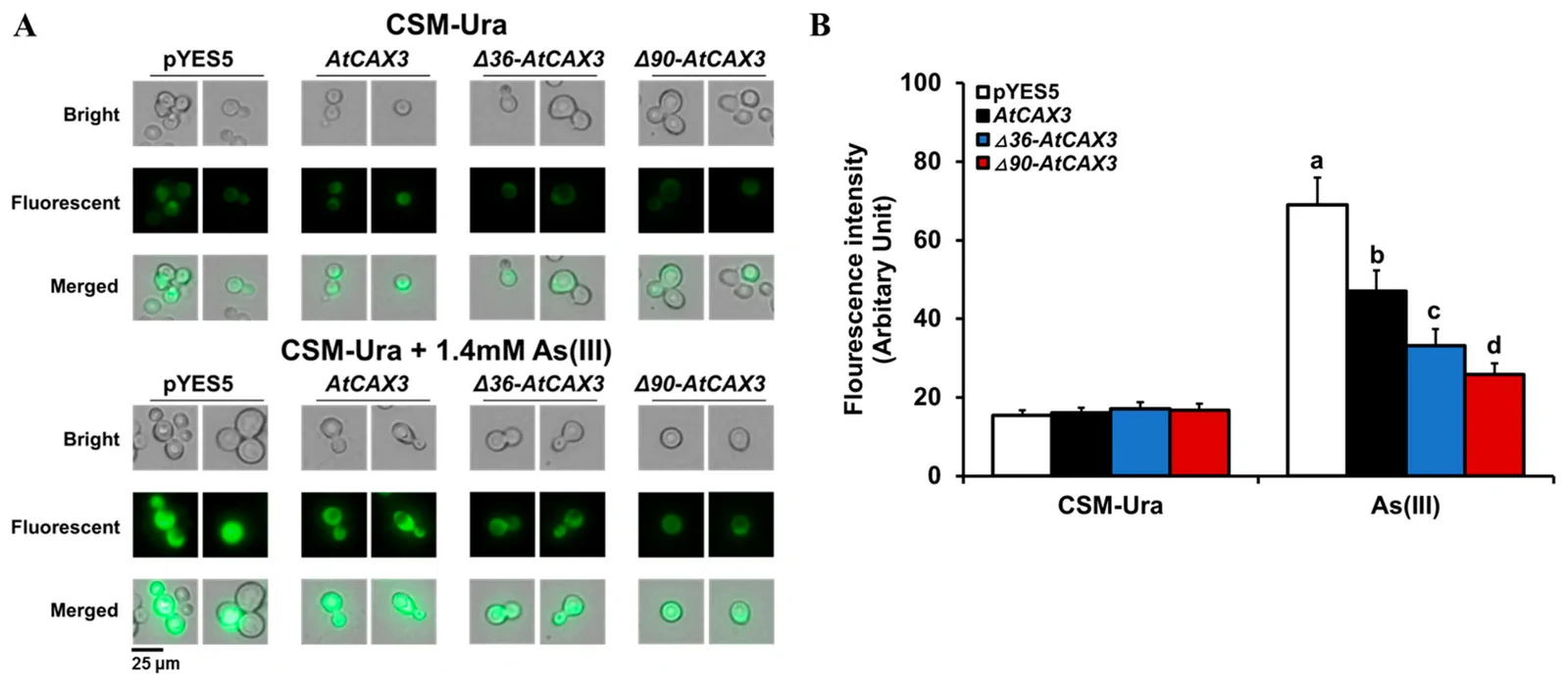
**ROS levels in yeast expressing *AtCAX3* and truncated constructs in response to BPA.** (**A**) Visualization of ROS (hydrogen peroxide) levels using the CM-H_2_DCFDA fluorescent probe. Control yeast (pYES5) and yeast expressing various *AtCAX3* constructs were grown for 24 h in CSM-Ura liquid medium with or without 30 ppm BPA. (**B**) Quantification of the ROS levels shown in (**A**). Fluorescence signals were quantified using ImageJ 1.54p(NIH, Bethesda, MD, USA). Data represent the mean ± SE of three independent biological experiments. Different lowercase letters above the bars indicate significant differences among different lines (*p* < 0.05).

It is well established that tolerance to environmental stresses is often negatively correlated with reactive oxygen species (ROS) accumulation, as toxic compounds such as BPA can induce excessive ROS production [23]. To investigate the relationship between BPA tolerance and ROS levels, hydrogen peroxide (H_2_O_2_) accumulation was visualized using the fluorescent probe CM-H_2_DCFDA in different yeast strains cultured in liquid medium (Figure 3A).

Fluorescence analysis showed that yeast strains with lower H_2_O_2_ accumulation exhibited higher tolerance to BPA. The reduction in ROS levels was observed in the following order: Δ90-AtCAX3 > Δ36-AtCAX3 > AtCAX3 > pYES5 (Figure 3B), which is similar to BPA tolerance patterns (Figure 2C). Notably, expression of AtCAX3 clearly reduced ROS accumulation compared with the pYES5 vector control, indicating that AtCAX3 contributes to oxidative stress alleviation during BPA exposure. These results suggest that AtCAX3 decreases ROS accumulation and that deletion of the autoinhibitory domains further strengthens this ROS-reducing effect.

### 2.3. Overexpression of N-Terminal Truncated AtCAX3 Enhances BPA Tolerance and Accumulation, and Ca^2+^ Accumulation in Arabidopsis

**Figure 4:**
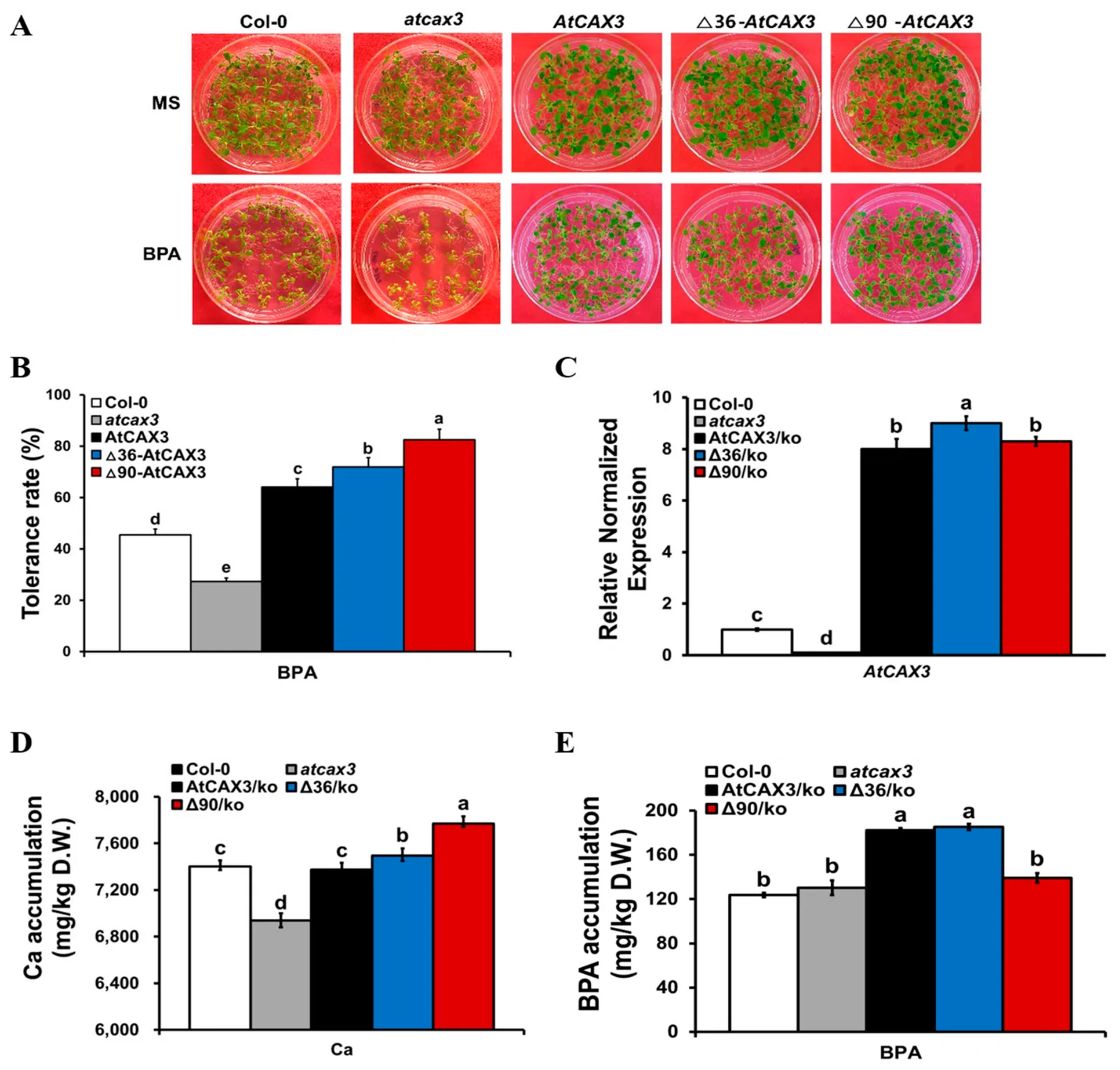
**BPA tolerance in Col-0 and *atcax3* Arabidopsis expressing various *AtCAX3* constructs.** (**A**) Representative photographs showing the growth of Col-0, *atcax3*, and *atcax3* lines expressing full-length *AtCAX3*, Δ36-*AtCAX3*, or Δ90-*AtCAX3* under BPA stress. (**B**) Relative tolerance rates (**C**) Transcript levels (**D**) Ca accumulation (**E**) BPA accumulation in Arabidopsis lines shown in (**A**). Data represent the mean of three independent experiments; error bars indicate SE. Different lowercase letters above the bars indicate significant differences among different lines (*p* < 0.05).

To evaluate the roles of AtCAX3 and its N-terminal truncated variants in BPA tolerance in plants, these constructs were overexpressed in Arabidopsis (Figure 4). As shown in Figure 4C, transgenic plants expressing *AtCAX3* constructs exhibited approximately 8- to 9-fold higher transcript levels than wild-type plants (Col-0), whereas *atcax3* mutant plants showed less than 10% of the expression level observed in Col-0. Under 30 ppm BPA treatment, tolerance levels were observed in the following order: Δ90-AtCAX3 > Δ36-AtCAX3 > AtCAX3 > Col-0 > *atcax3* (Figure 4A,B). These results were consistent with those obtained in yeast (Figure 2), suggesting that AtCAX3 enhances BPA tolerance in Arabidopsis and that deletion of the autoinhibitory domains (Δ36 and Δ90) further strengthens this tolerance.

Because AtCAX3 is involved in Ca^2+^ uptake and vacuolar sequestration/accumulation [28], endogenous Ca^2+^ levels were subsequently examined. As shown in Figure 4D, expression of AtCAX3 restored Ca^2+^ levels in *atcax3* mutant plants to those of Col-0, whereas *atcax3* mutants exhibited reduced Ca^2+^ accumulation. Furthermore, Ca^2+^ levels were significantly increased in Δ90-AtCAX3- and Δ36-AtCAX3-expressing plants, suggesting that deletion of the autoinhibitory domains enhances Ca^2+^ sequestration and accumulation, thereby contributing to increased BPA tolerance.

To further investigate the relationship between BPA tolerance and BPA accumulation, BPA contents were analyzed in wild-type and transgenic plants. As shown in Figure 4E, BPA accumulation in full-length AtCAX3- and Δ36-AtCAX3-expressing plants was higher than that in Col-0 plants. These results suggest that expression of AtCAX3, particularly full-length and Δ36-AtCAX3, enhances both BPA tolerance and BPA accumulation. In contrast, the low BPA tolerance of *atcax3* mutants may result from the absence of effective detoxification or sequestration mechanisms mediated by AtCAX3. However, the reason why Δ90-AtCAX3-expressing plants did not exhibit increased BPA accumulation remains unclear. In short, BPA tolerance and Ca^2+^ levels do not match BPA accumulation, suggesting that BPA tolerance is not related to BPA accumulation in these plants.

### 2.4. Expressions of Ca Exporters Were Reduced and That of Ca Importers Were Enhanced in N-Terminal Truncated AtCAX3-Expressing Arabidopsis

To elucidate the mechanism underlying the increased Ca^2+^ accumulation observed in various AtCAX3-expressing lines under BPA treatment (Figure 4D), the expression levels of several Ca^2+^ transporters were analyzed, including the Ca^2+^ exporters *AtACA8* and *AtACA10* (Ca^2+^-ATPases) and the Ca^2+^ importers *AtCNGC10*, *AtMCA1*, and *AtGLR3.3*.

**Figure 5:**
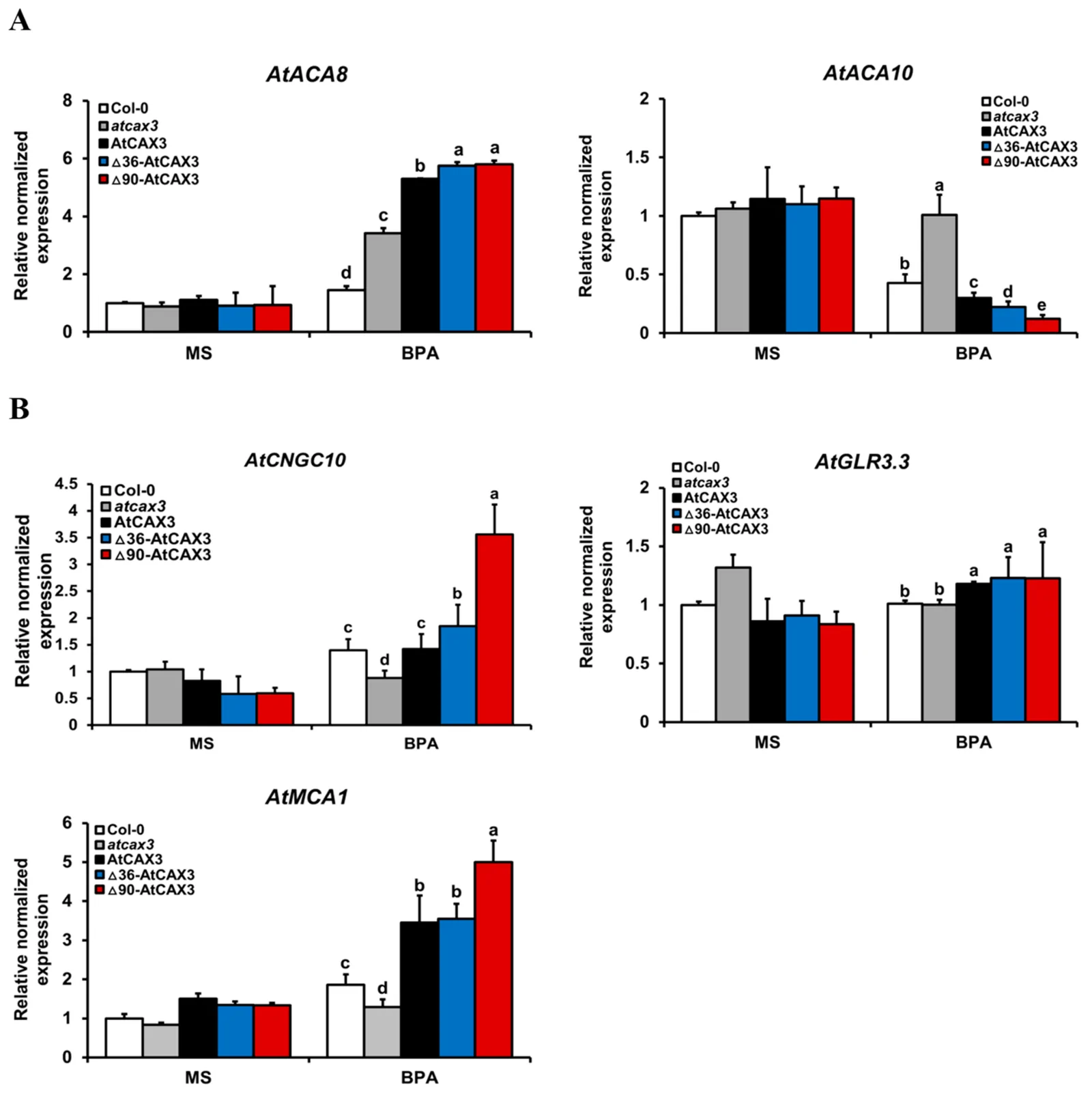
**Relative expression of Ca^2+^ transporter genes in Col-0 and various *atcax3* Arabidopsis lines under BPA stress.** (**A**) qRT-PCR analysis of Ca^2+^ exporters *AtACA8* and *AtACA10*. (**B**) qRT-PCR analysis of Ca^2+^ importers *AtCNGC10*, *AtGLR3.3*, and *AtMCA1*. Seedlings were grown for 21 days on 1/2 MS agar medium with or without 30 ppm BPA. Total RNA was extracted from 0.1 g of whole seedlings. Data represent the mean ± SE of three independent biological experiments. Different lowercase letters above the bars indicate significant differences among different lines (*p* < 0.05).

As shown in Figure 5A, expression of the Ca^2+^ exporter *AtACA10* was significantly reduced following BPA treatment, with the degree of reduction occurring in the order: Δ90-AtCAX3 > Δ36-AtCAX3 > AtCAX3 > Col-0 > *atcax3*. In contrast, *AtACA8* expression exhibited a different response pattern under BPA treatment, showing higher expression in the order: Δ90-AtCAX3 = Δ36-AtCAX3 > AtCAX3 > *atcax3* > Col-0.

The expression levels of the Ca^2+^ importers *AtCNGC10* and *AtMCA1* were significantly increased in response to BPA treatment and followed the order: Δ90-AtCAX3 > Δ36-AtCAX3 = AtCAX3 > Col-0 > *atcax3*. However, expression of *AtGLR3.3* was not significantly altered by BPA treatment compared with untreated controls.

These results suggest that the increased Ca^2+^ accumulation observed in various *AtCAX3*-expressing Arabidopsis lines is associated with downregulation of the Ca^2+^ exporter *AtACA10* and upregulation of the Ca^2+^ importers *AtCNGC10* and *AtMCA1*.

### 2.5. N-Terminal Truncated AtCAX3 Expression Reduces ROS Accumulation and Enhances Antioxidant Enzyme Activities in Arabidopsis

**Figure 6:**
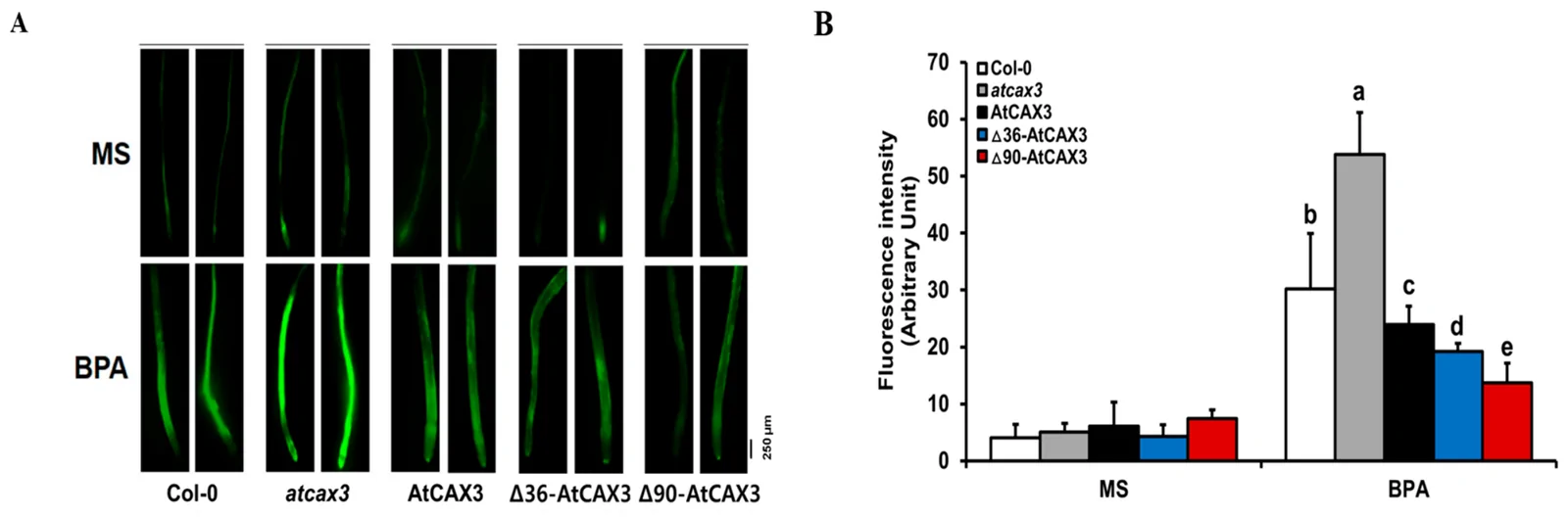
**ROS levels in Col-0 and *atcax3* Arabidopsis expressing various *AtCAX3* in response to BPA.** (**A**) Visualization of ROS (hydrogen peroxide) in Arabidopsis root tips using the CM-H_2_DCFDA probe. Tested lines include Col-0, *atcax3*, and *atcax3* complemented with *AtCAX3*, Δ36-*AtCAX3*, or Δ90-*AtCAX3*. (**B**) Quantification of the fluorescence signals shown in (**A**) using ImageJ. Plants were grown for 21 days on 1/2 MS agar medium with or without 30 ppm BPA. Data represent the mean of three independent experiments; error bars indicate SE. Different lowercase letters above the bars indicate significant differences among different lines (*p* < 0.05).

To investigate the relationship between ROS and Ca^2+^ under BPA stress, ROS levels were analyzed in various AtCAX3-expressing Arabidopsis lines. As shown in Figure 6A,B, hydrogen peroxide levels, visualized using CM-H_2_DCFDA fluorescence, were similar among Col-0, *atcax3*, and AtCAX3 transgenic plants under control conditions (1/2MS medium). However, following BPA treatment, ROS accumulation was observed in the following order: *atcax3* > Col-0 > AtCAX3 > Δ36-AtCAX3 > Δ90-AtCAX3. These results suggest that expression of AtCAX3 enhances BPA tolerance primarily through reduction in ROS accumulation associated with increased Ca^2+^ accumulation, rather than through reduction in internal BPA levels (Figure 4).

**Figure 7:**
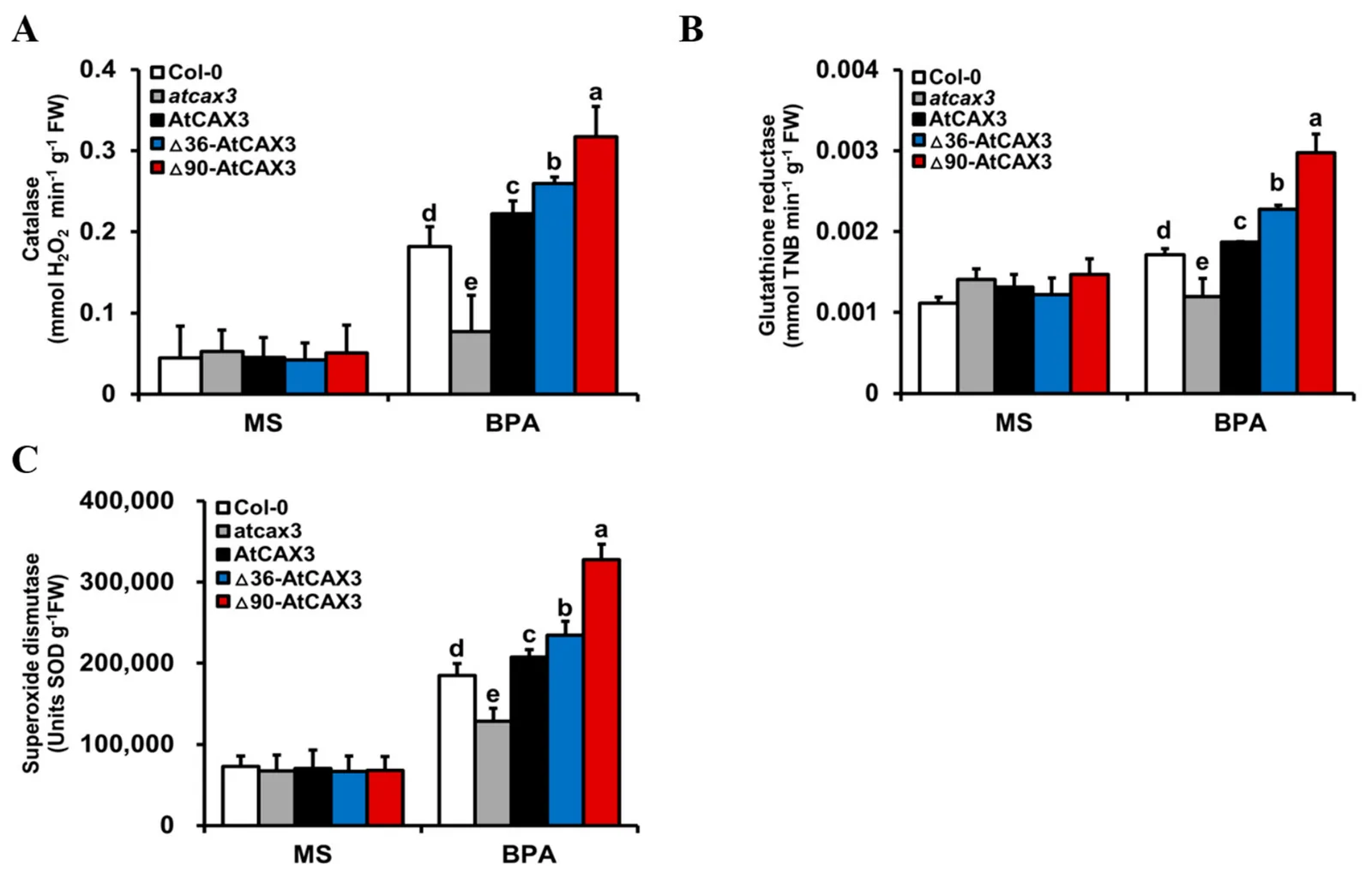
**Antioxidant enzyme activities in Col-0 and various *atcax3* Arabidopsis lines under BPA stress.** (**A**) Catalase (CAT), (**B**) glutathione reductase (GR), and (**C**) superoxide dismutase (SOD) activities were measured in Col-0, *atcax3*, and complemented lines. Plants were grown for 21 days on 1/2 MS agar medium with or without 30 ppm BPA. Enzyme activities were measured using extracts from 0.3 g of whole seedlings. Data are the mean of three independent experiments; error bars indicate SE. Different lowercase letters above the bars indicate significant differences among different lines (*p* < 0.05).

To determine whether the reduced ROS levels in AtCAX3 transgenic lines were associated with enhanced antioxidant defense systems, the activities of catalase (CAT), glutathione reductase (GR), and superoxide dismutase (SOD) were measured. As shown in Figure 7, no significant differences in CAT, GR, or SOD activities were detected among the different lines under control conditions. In contrast, BPA treatment significantly increased the activities of these antioxidant enzymes in AtCAX3 transgenic plants compared with Col-0 and *atcax3* plants. The induction pattern of antioxidant enzyme activities was as follows: Δ90-AtCAX3 > Δ36-AtCAX3 > AtCAX3 > Col-0 > *atcax3*.

This trend was negatively correlated with ROS accumulation (Figure 6) and positively correlated with BPA tolerance (Figure 4). Collectively, these findings suggest that overexpression of AtCAX3, particularly its N-terminal truncated forms, reduces ROS accumulation by enhancing antioxidant enzyme activities, most likely through increased Ca^2+^ accumulation and Ca^2+^-mediated stress signaling.

## 3. Discussion

### 3.1. AtCAX3 Overexpression Enhances BPA Tolerance and Accumulation

Previously, we demonstrated that AtCAX3 is involved in Cd tolerance and that the deletion of its autoinhibitory domain (Δ90) further enhances this tolerance [38]. In the present study, we show that *AtCAX3* overexpression confers enhanced tolerance to BPA in Arabidopsis. Specifically, the deletion of the N-terminal autoinhibitory domains (Δ36 and Δ90) in AtCAX3 significantly improves BPA tolerance. The *AtCAX3*- and Δ36-*AtCAX3*-expressing Arabidopsis lines exhibited significantly increased BPA accumulation, suggesting that AtCAX3 may contribute to BPA accumulation in plants (Figure 4E). The combination of increased BPA accumulation and enhanced BPA tolerance in these lines may reflect an increased capacity to mitigate BPA toxicity, thereby allowing greater BPA accumulation without causing severe physiological damage. In contrast, the *atcax3* mutant displayed BPA levels similar to control plants and lower BPA tolerance. This discrepancy suggests that internal BPA concentration alone is not the sole determinant of toxicity. In full-length *AtCAX3*- and Δ36-*AtCAX3*-expressing plants, enhanced Ca^2+^ levels and/or signaling may activate vacuolar sequestration mechanisms, potentially involving ABC transporters or GST-mediated conjugation, thereby facilitating the compartmentalization of BPA away from sites of active metabolism. In contrast, the *atcax3* mutant may have a reduced Ca^2+^-dependent protective response, potentially resulting in increased BPA-induced cellular toxicity.

Notably, the Δ90-*AtCAX3* line showed the highest tolerance but did not exhibit increased BPA accumulation. This unique phenotype could be explained by two hypotheses. First, the hyper-activation of Ca^2+^ signaling in Δ90 lines may strongly induce the expression of efflux pumps that actively transport BPA out of the root cells back into the medium. Second, the downstream metabolic pathways that degrade or transform BPA into undetectable metabolites might be uniquely upregulated in the Δ90 line due to its superior Ca^2+^/antioxidant status (Figure 4 and Figure 7).

### 3.2. Mitigation of ROS via AtCAX3-Induced Antioxidant Enzymes

The superior BPA tolerance observed in various *AtCAX3*-expressing plants, compared with control plants, can be attributed to lower ROS levels (Figure 6). This reduction in BPA-induced ROS by AtCAX3 may be explained by the enhanced activity of antioxidant enzymes observed across the various *AtCAX3*-expressing lines (Figure 7). This increased enzymatic activity likely results from elevated Ca^2+^ levels in *AtCAX3*-expressing plants, as antioxidant enzyme activities are well-known to be enhanced by Ca^2+^ during plant responses to abiotic stresses, including heat [36], arsenic [37], and atrazine stress [39].

Mechanistically, AtCAX3-mediated Ca^2+^ homeostasis may modulate Ca^2+^ signaling pathways that activate ROS-scavenging enzymes through various Ca^2+^-dependent proteins. For example, increased Ca^2+^ reduces H_2_O_2_ production by facilitating calmodulin (CaM) binding to catalase, as observed in tobacco [40]. In rice, Ca^2+^/CaM-dependent protein kinase (CCaMK) mediates ABA-induced gene expression and enhances SOD and CAT activities to alleviate drought-induced ROS accumulation [41]. Similarly, Brassinosteroid-induced cytosolic Ca^2+^ activates CCaMK in maize, subsequently enhancing the expression and activity of ascorbate peroxidase (APX) and SOD [42]. Furthermore, in the marine alga *Ectocarpus siliculosus*, copper stress elevates cytosolic Ca^2+^ levels, thereby increasing the expression of SOD, APX, GR, and peroxiredoxin (PRX) through CaMK-, PKA-, and CBLK-mediated pathways [43]. Based on the proposed function of AtCAX1 [44,45], we propose that AtCAX3 disrupts the positive feedback loop between ROS generation and Ca^2+^ spikes, leading to a decrease in ROS levels.

### 3.3. Regulation of Ca^2+^ Transporters and Intracellular Homeostasis by AtCAX3

To understand the mechanism underlying enhanced Ca^2+^ accumulation in various *AtCAX3*-expressing plants, expression analysis of several endogenous Ca^2+^ transporters was performed (Figure 5). The results revealed a significant down-regulation of the Ca^2+^ exporter *AtACA10* and up-regulation of the Ca^2+^ importers *AtCNGC10* and *AtMCA1* in response to BPA, collectively contributing to elevated intracellular Ca^2+^ levels. Conversely, the lower Ca^2+^ levels observed in *atcax3* mutants may result from the opposite transcriptional profile, namely the up-regulation of *AtACA10* and down-regulation of *AtCNGC10* and *AtMCA1*. These results suggest that *AtCAX3* expression may influence the feedback regulation of these Ca^2+^ transporters, although the precise underlying molecular mechanism remains to be elucidated.

Beyond coordinating other transporters, the vacuolar cation exchanger AtCAX3 itself likely contributes directly to Ca^2+^ accumulation in transgenic plants by driving influx into the vacuole. Although elevated intracellular Ca^2+^ levels are a common secondary messenger response in plants exposed to environmental stimuli [46,47], prolonged, uncontrolled increases in cytosolic Ca^2+^ can induce severe cytotoxicity. Therefore, plants employ a tightly regulated network of pumps, channels, and exchangers to maintain strict Ca^2+^ homeostasis among the cytosol, organelles, and apoplast during xenobiotic stress.

### 3.4. Enhanced Ca^2+^ Homeostasis and BPA Tolerance by N-Terminal Truncated AtCAX3 Variants

Previous biochemical studies have established that the removal of the N-terminal regulatory region (NRR) in AtCAX1 and AtCAX3 removes an autoinhibitory mechanism, thereby enhancing vacuolar Ca^2+^ transport activity, leading to increased Ca^2+^ accumulation and enhanced stress tolerance [48,49]. Our results are highly consistent with these findings, as Δ90-AtCAX3- and Δ36-AtCAX3-expressing yeast and Arabidopsis lines exhibited significantly enhanced BPA tolerance compared with full-length AtCAX3-expressing lines (Figure 2 and Figure 4B). This enhanced tolerance strongly correlated with higher absolute Ca^2+^ accumulation within the tissues (Figure 4D). Overall, the degree of BPA tolerance and total Ca^2+^ levels among the studied lines followed a clear, hierarchical order: Δ90-AtCAX3 > Δ36-AtCAX3 > AtCAX3 > Col-0 > atcax3 (Figure 4B,D), confirming that optimizing vacuolar Ca^2+^ sequestration capacity is an effective strategy for mitigating BPA toxicity in plants. Therefore, it would be valuable to investigate whether other CAX family members that contribute to elevated intracellular Ca^2+^ levels also enhance BPA tolerance, although, to date, no CAX proteins other than AtCAX3 have been reported to be involved in BPA tolerance.

## 4. Materials and Methods

### 4.1. Plasmid Construction

To construct overexpression vectors for *AtCAX3*, Δ36-*AtCAX3*, and Δ90-*AtCAX3* in *Saccharomyces cerevisiae*, *AtCAX3* cDNA was first amplified from wild-type *Arabidopsis thaliana* using specific primers containing *Spe*I and *Hind*III sites (Table S1). The PCR product was then double-digested with *Spe*I and *Hind*III and cloned into the pYES5 vector [50] using T4 DNA ligase (TAKARA, Tokyo, Japan).

For overexpression in *Arabidopsis*, the Gateway cloning system was used. The *AtCAX3* gene was amplified from wild-type *Arabidopsis* cDNA using primers containing *attB1* and *attB2* sites (Table S1), and cloned into the pDONR221™ entry vector using Gateway BP Clonase (Invitrogen, Carlsbad, CA, USA). The entry clone was then recombined with the pMDC83 destination vector containing the CaMV 35S promoter and green fluorescent protein (GFP) using Gateway LR Clonase (Invitrogen, USA), resulting in constructs expressing *AtCAX3*, Δ36-*AtCAX3*, and Δ90-*AtCAX3*.

### 4.2. Transformation

For yeast transformation, *S. cerevisiae* (strain ATCC 4001531) was transformed with pYES5, pYES5-*AtCAX3*, pYES5-Δ36-*AtCAX3*, and pYES5-Δ90-*AtCAX3* using the lithium acetate/polyethylene glycol (LiAc/PEG) method [51]. Salmon sperm DNA (10 mg/mL, Sigma, St. Louis, MI, USA) was used as carrier DNA. Transformed yeast cells were plated on CSM-Ura selection medium and incubated for 3–5 days to confirm transformants.

For *Arabidopsis* transformation, the constructs pMDC83, pMDC83-*AtCAX3*, pMDC83-Δ36-*AtCAX3*, and pMDC83-Δ90-*AtCAX3* were first introduced into *Agrobacterium tumefaciens* strain EHA105 using the freeze–thaw method. These strains were then used to transform *atcax3* knockout *Arabidopsis* plants via the floral dipping method [52]. The *atcax3* mutant line (line *atcax3-1* supplied from Dr. Hirsch, Baylor College of Medicine, Houston, TX, USA) carries a BASTA resistance gene, whereas the transformants were selected on medium containing 15 μg/mL BASTA and 20 μg/mL hygromycin (Hyg) due to their Hyg resistance gene. Transgenic lines were confirmed by genetic analysis and expression verification.

### 4.3. Semi-Quantitative RT-PCR and qRT-PCR

Arabidopsis seedlings were grown for three weeks on 1/2 MS medium (10 g/L sucrose, 6 g/L agar, 2.207 g/L MS salts) containing antibiotics. Then, 0.1 g of seedlings was harvested, snap-frozen in liquid nitrogen, and dried. RNA was extracted using an RNA extraction kit (FAVROGEN, Pingtung City, Taiwan). Total RNA was quantified using a Biospec-nano spectrophotometer (SHIMADZU BIOTECH, Kyoto, Japan), and 2 μg of RNA was reverse-transcribed to cDNA using NEXscript™ Oligo dT RT 2X Master Mix (Genes lab., Seongnam, Republic of Korea). To verify *AtCAX3* overexpression, semi-quantitative RT-PCR was performed using *AtCAX3*-specific primers (Table S1) under the following conditions: 95 °C for 1 min (denaturation), followed by 35 cycles of 95 °C for 30 s, 57 °C for 30 s (annealing), and 72 °C for 1 min 45 s (extension). *AtACTIN* was used as the internal control. qRT-PCR analysis was performed using the CFX Connect™ Real-Time PCR Detection System (Bio-Rad, Hercules, CA, USA). Expression levels were normalized to *AtACTIN*. Each reaction included 2 μL cDNA, 10 μL SsoAdvanced™ Universal SYBR^®^ Green Supermix (Bio-Rad, Hercules, CA, USA), 6 μL DEPC water, and 10 pmol each of forward and reverse primers. All experiments were conducted in triplicate, including three independent biological replicates.

### 4.4. Determination of Yeast Tolerance to BPA

Yeast lines expressing pYES5, *AtCAX3*, Δ36-*AtCAX3*, or Δ90-*AtCAX3* were grown in CSM-Ura liquid medium to the exponential phase. Cultures were adjusted to OD_600_ of 0.3, then serially diluted to 10^−1^, 10^−2^, and 10^−3^. Diluted cells were plated on YPD medium containing various concentrations of BPA and incubated for 2 days at 30 °C. Resistance was evaluated by comparing colony number and size. This experiment was independently repeated three times. Growth analysis was conducted by adjusting overnight cultures to OD_600_ of 0.1 and incubating them in 20 mL CSM-Ura liquid medium with varying concentrations of BPA at 30 °C and 200 rpm for 24 h. OD_600_ values were measured at 6, 18, and 24 h. All experiments were independently repeated three times.

### 4.5. Visualization of ROS Levels in Yeast

The CM-H_2_DCFDA fluorescent probe (Thermo Fisher, Waltham, MA, USA) was used to assess ROS levels in yeast. A modified version of the standard enzymatic lysis method was used [53]. Yeast cultures at OD_600_ = 0.9 were subjected to enzymatic lysis to produce spheroplasts. These were resuspended in 10 μM CM-H_2_DCFDA and incubated in the dark for 30 min, followed by PBS washing. After resuspension in PBS, cells were imaged using a fluorescence microscope (OLYMPUS IX73, Tokyo, Japan). ROS levels were quantified using ImageJ software (version: 1.54p, NIH, Bethesda, MD, USA), analyzing at least 8 yeast cells per line in three independent biological experiments.

### 4.6. Determination of Tolerance to BPA in Arabidopsis

Wild-type *Arabidopsis* Col-0, *atcax3* mutant, and *atcax3* background lines overexpress ing *AtCAX3*, Δ36-*AtCAX3*, or Δ90-*AtCAX3* (T3 homozygous) were used. Sterilized seeds were sown on 1/2 MS medium or 1/2 MS medium supplemented with 30 ppm BPA. Twenty-five seeds per line were grown under a 16 h light/8 h dark photoperiod for 21 days. Phenotypes were recorded, and seedling weights were measured. Tolerance was calculated by dividing the fresh weight of BPA-treated plants by that of control (non-treated) plants. Experiments were repeated three times.

### 4.7. Measurement of Ca Concentration

Ca accumulation was measured using transgenic plants grown for 21 days on 1/2 MS agar medium. The harvested seedlings were rinsed with distilled water for Ca measurements. The Ca accumulation was quantified using Inductively Coupled Plasma-Optical Emission Spectrometry (ICP-OES, iCAP 7400 DUO, Thermo Fisher Scientific, Waltham, MA, USA) at a wavelength of 370.60 nm. Three biological replicates were independently carried out.

### 4.8. Quantification of Bisphenol A

Wild-type (Col-0) *Arabidopsis* seedlings were grown vertically for 3 weeks on half-strength Murashige and Skoog (1/2 MS) agar medium supplemented with 30 ppm BPA. Ground plants (1 mg) were extracted with 1 mL of methanol (Sigma-Aldrich, Seoul, Republic of Korea), and the resulting methanolic supernatants were analyzed. An Acquity UPLC-MS/MS system (Waters, Milford, MA, USA) equipped with an electrospray ionization (ESI) source was used for the analysis. Chromatographic separation was performed using an Acquity UPLC BEH C18 column (1.7 µm, 2.1 × 100 mm). The mobile phase consisted of distilled water (solvent A) and methanol (solvent B) at a flow rate of 0.4 mL/min. The gradient elution program was set as follows: 0 min, 80:20 (A:B); 4.5 min, 35:65; 5.6 min, 0:100; 7 min, 0:100; 7.01 min, 80:20; and 9 min, 80:20. Multiple reaction monitoring (MRM) was performed in the negative ion mode. The target MRM transitions were *m*/*z* 227.1 → 212.0 for BPA and *m*/*z* 241.3 → 223.2 for BPA-d16 (internal standard). The calibration curve was constructed over a concentration range of 10–500 ng/mL. Data are presented as the average of three independent biological replicates.

### 4.9. Visualization of ROS Levels in Arabidopsis

To visualize ROS in planta, seedlings were grown on 1/2 MS medium (control) or medium containing 30 ppm BPA for 21 days. Harvested plants were incubated in 10 μM CM-H_2_DCFDA in the dark for 30 min and washed 3–5 times with distilled water. Fluorescence was captured using a Leica MZ10F microscope (Leica Microsystems, Wetzlan, Germany) with excitation at 485 nm and emission at 510–530 nm. Fluorescence intensity was quantified using ImageJ for at least 10 seedlings per line.

### 4.10. Measurement of Antioxidant Enzyme Activities in Arabidopsis

The activities of ROS-scavenging (antioxidant) enzymes were assayed using crude plant extracts. Briefly, 200 mg of frozen leaves or roots from 3-week-old plants were ground in liquid nitrogen and homogenized in 1.2 mL of extraction buffer (pH 7.8, 200 mM potassium phosphate, 0.1 mM EDTA). The homogenates were centrifuged at 15,000× *g* for 20 min at 4 °C. The resulting supernatants were collected to determine the activities of glutathione reductase (GR), catalase (CAT), and superoxide dismutase (SOD), following the protocols described by Bahmani et al. [54]. Three independent biological experiments were performed, each with three technical replicates.

### 4.11. Statistical Analysis

Data analysis was performed using SAS software (version 9.1). All data are presented as the means of three independent experiments. Statistical significance was determined using Tukey’s honest significant difference (HSD) test for multiple comparisons (*p* < 0.05).

## 5. Conclusions

In conclusion, our study demonstrates that the N-terminal truncation of AtCAX3 removes autoinhibition, leading to enhanced vacuolar Ca^2+^ accumulation. This altered Ca^2+^ homeostasis orchestrates a tripartite defense mechanism against BPA stress: it remodels the expression of endogenous Ca^2+^ transporters to sustain Ca^2+^ levels, boosts the enzymatic antioxidant system to scavenge ROS, and potentially modulates BPA sequestration or metabolism. These findings may contribute to the development of BPA-remediating plants and crops resilient to xenobiotic and BPA contamination, while providing new insights into the role of CAX proteins in plant responses to abiotic and BPA stresses.

## Data Availability

The data supporting the findings of this study are available within the paper and its Supplementary Information files.

## Supplementary Materials

The following supporting information can be downloaded at https://www.mdpi.com/article/10.3390/plants15193042/s1, Table S1: Sequences of the primers used in this study.
