# Supplementary material for: Molecular Mechanism of AtCAX3-Induced BPA Tolerance in Plants
Source: Plants (Basel). 2026 Oct 5;15(19):3042. doi: 10.3390/plants15193042 (PMC13666901; doi:10.3390/plants15193042)
Supplement: Supplementary file 1 [file plants-15-03042-s001.zip › plants-4559934-supplementary.pdf]

Table1. Primer sequences used in this study.

Primer	Description	Sequence (5′–3′)
*AtCAX3*_Full_ *Spe*I_F	Yeast transformation	ATCGGTACCATGGGAAGTATCGTGGAGCC
*AtCAX3*_36_ *Spe*I_F	Yeast transformation	ATCGGTACCATGTCTTCATCGTCGCTAAGG
*AtCAX3*_90_*Spe*I_F	Yeast transformation	ATCGGTACCATGTACAACTACGGTCGTCC
*AtCAX3*_*Hind*III_R	Yeast transformation	ATCGAGCTCTTAAGCTGAGAAAACTTCTCC
*AtCAX3*_attB1_Full-F	Plant Transformation	GGGGACAAGTTTGTACAAAAAAGCAGGCTTAATGGGAAGTATCGTGGAGCC
*AtCAX3*_attB1_36-F	Plant transformation	GGGGACAAGTTTGTACAAAAAAGCAGGCTTAATGTCTTCATCGTCGCTAAGG
*AtCAX3*_attB1_90-F	Plant transformation	GGGGACAAGTTTGTACAAAAAAGCAGGCTTAATGTACAACTACGGTCGTCC
*AtCAX3*_attB2_R	Plant transformation	GGGGACCACTTTGTACAAGAAAGCTGGGTTAGCTGAGAAAACTTCTCCCA
RT_*AtActin*_F	RT-qPCR	GCAGAGCGGGAAATTGTAAG
RT_*AtActin*_R	RT-qPCR	TTCTCGATGGAAGAGCTGGT
RT_*AtCAX3*_F	RT-qPCR	ATGCTGGAGCCATCATTTTC
RT_*AtCAX3*_R	RT-qPCR	AGGATCCACGCAACGATAAC
RT_*AtACA8*_F	RT-qPCR	CTCTTGGAGCATTGGCTTTG
RT_*AtACA8*_R	RT-qPCR	TCATGCACTTCATGCTCCAG
RT_*AtACA10*_F	RT-qPCR	GCACGCCCAAGCAATAAGAG
RT_*AtACA10*_R	RT-qPCR	TCTGCTCCTGTCCGATACCA
RT_*AtCNGC10*_F	RT-qPCR	CCAAGTCACTACGCGATGAA
RT_*AtCNGC10*_R	RT-qPCR	GGTGGGTTTTTGAGGTAGCA
RT_*AtGLR3.3*_F	RT-qPCR	GGCTAATGAAGAACGCATG
RT_*AtGLR3.3*_R	RT-qPCR	GCATGGAGGAAGAGTCATGA
RT_*AtMCA1*_F	RT-qPCR	TGTTGCTCGGAACCTTCTCT
RT_*AtMCA1*_R	RT-qPCR	AATCCTCCCGTGATGTTCAG
